# Liposome-Based Co-Immunotherapy with TLR Agonist and CD47-SIRPα Checkpoint Blockade for Efficient Treatment of Colon Cancer

**DOI:** 10.3390/molecules28073147

**Published:** 2023-03-31

**Authors:** Rui Chang, Xiaohong Chu, Jibing Zhang, Rongrong Fu, Changshun Feng, Dianlong Jia, Rui Wang, Hui Yan, Guangyong Li, Jun Li

**Affiliations:** School of Pharmaceutical Sciences, Liaocheng University, Liaocheng 252059, China

**Keywords:** imiquimod, liposome, Fc-CV1, immunotherapy, colon cancer

## Abstract

Antitumor immunity is an essential component of cancer therapy and is primarily mediated by the innate immune response, which plays a critical role in initiating and shaping the adaptive immune response. Emerging evidence has identified innate immune checkpoints and pattern recognition receptors, such as CD47 and Toll-like receptor 7 (TLR7), as promising therapeutic targets for cancer treatment. Based on the fusion protein Fc-CV1, which comprises a high-affinity SIRPα variant (CV1), and the Fc fragment of the human IgG1 antibody, we exploited a preparation which coupled Fc-CV1 to imiquimod (TLR7 agonist)-loaded liposomes (CILPs) to actively target CT26. WT syngeneic colon tumor models. In vitro studies revealed that CILPs exhibited superior sustained release properties and cell uptake efficiency compared to free imiquimod. In vivo assays proved that CILPs exhibited more efficient accumulation in tumors, and a more significant tumor suppression effect than the control groups. This immunotherapy preparation possessed the advantages of low doses and low toxicity. These results demonstrated that a combination of immune checkpoint blockade (ICB) therapy and innate immunity agonists, such as the Fc-CV1 and imiquimod-loaded liposome preparation utilized in this study, could represent a highly effective strategy for tumor therapy.

## 1. Introduction

The global incidence of cancer continues to rise annually, posing a serious threat to the health of the world’s population and placing a heavy burden on healthcare systems. According to a World Health Organization report, in 2020, 19.29 million new cases of cancer and 9.96 million cancer deaths occurred worldwide [1]. However, with the continuous advancement in clinical diagnosis, surgery, radiotherapy, and chemotherapy, the overall survival and quality of life of cancer patients have improved significantly. Notably, immunotherapy, which originated with the Coley vaccine in the 1890s [2], predates both radiotherapy and chemotherapy, and has made significant progress in the last decade, benefiting cancer patients.

Tumor immunotherapy has been recognized for its excellent biocompatibility and high specificity, and is capable of inducing immune responses to eliminate tumor cells [3,4,5]. Two main strategies for tumor immunotherapy are immune enhancement and immune normalization [6]. The patterns of immune enhancement mainly consist of cytokine therapy, tumor therapeutic vaccines, and monoclonal antibodies. Toll-like receptors (TLRs) are considered potential targets for cancer immunotherapy due to their role in the activation of the innate immune system. TLR agonists have been developed as an immune adjuvant, and a variety of TLR agonists are undergoing clinical trials [7]. Monophosphoryl lipid A and imiquimod, TLR4 and TLR7 agonists, respectively, have received approval from the Food and Drug Administration for clinical application [7,8,9]. Imiquimod, a TLR7 agonist [9], activates immune responses and enhances anti-tumor activity by triggering TLR7 [10,11].

The immune normalization strategy is represented by immune checkpoint blockade (ICB) drugs which regulate the defective adaptive tumor immune response. Since the first checkpoint inhibitor, ipilimumab, was approved for clinical application in the USA in 2011, immune checkpoint inhibitors, such as PD1/PDL1 inhibitors, have made a breakthrough in cancer immunotherapy [12,13,14]. Except for adapted immune checkpoints similar to PD1/PDL1, some innate immune checkpoints, such as SIRPα-CD47, have also attracted much attention and become areas of significant interest in antibody drug development [15,16,17].

The SIRPα-CD47 checkpoint was first identified in 1999 [18,19]. SIRPα is a CD47 receptor expressed in central nervous system neurons [20] and myeloid cells, including monocytes, macrophages, granulocytes, and dendritic cells. CD47 is a “self-labeling molecule” expressed on nearly all normal cells and overexpressed on most tumor cells [21]. On the surface of tumor cells, CD47 binds to SIRPα, inhibiting macrophage-mediated phagocytosis, which is an important means of tumor immune escape [22]. It has been shown that the phagocytosis of macrophages within the tumor microenvironment can be enhanced when the binding of CD47 and SIRPα was blocked [20,21,22,23,24,25]. Therefore, immune checkpoint inhibitors that block the CD47/SIRPα pathway have been developed and applied in cancer therapy [26].

In this study, we used a combination of imiquimod and fusion protein (Fc-CV1) to treat colon cancer. CV1 is a modified human recombinant SIRPα protein that has a 50,000-fold higher affinity for CD47 than the original protein [27]. Fc-CV1 was constructed using “Knobs-into-holes” technology, based on the CV1 monomer and Fc fragment of human IgG1. To overcome the limitations of poor therapeutic efficacy and systemic toxicity associated with imiquimod [28,29], we prepared nanoliposomes through the ethanol injection method. The nanoliposomes have the characteristics of low toxicity in drug delivery [30,31]. Subsequently, imiquimod was encapsulated in liposomes which surface conjugated Fc-CV1. Finally, this novel nano-preparation can actively deliver TLR7 agonists to tumor tissues, and has the dual functions as an ICB drug and a TLR agonist. It was used to treat the CD47^+^ CT26.WT syngeneic colon tumor model [32].

## 2. Results and Discussion

### 2.1. Characterization of LPs (Blank Liposomes), ILPs (Non-CD47-Targeted Imiquimod-Encapsulated Liposomes), CLPs (CD47 Targeted Liposomes), and CILPs (Coupled Fc-CV1 to Imiquimod (TLR7 agonist)-Loaded Liposomes)

**Figure 1:**
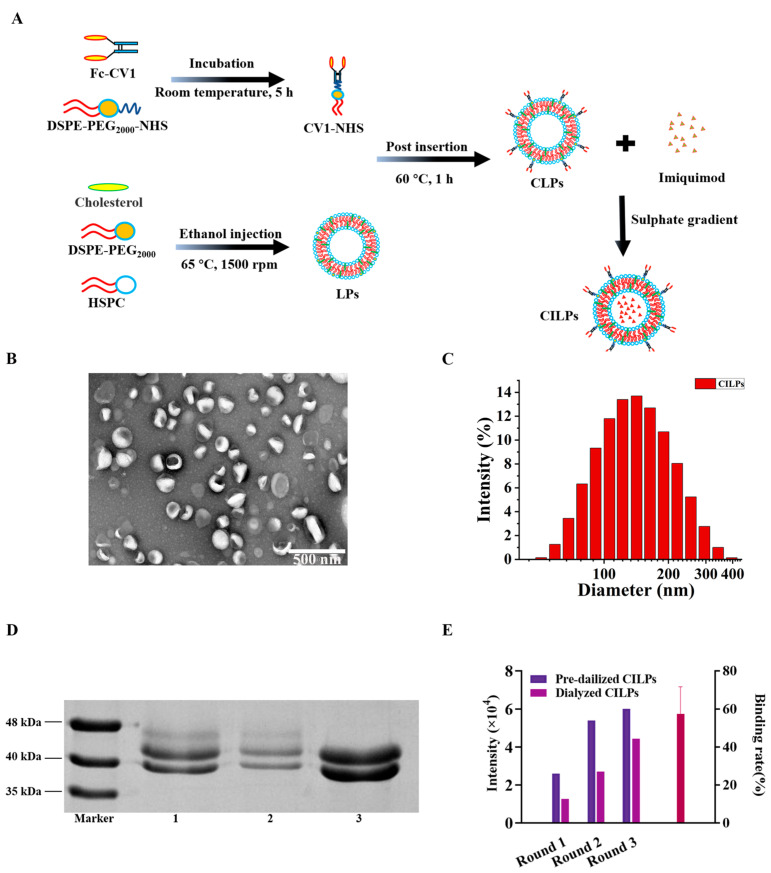
(**A**) Schematic illustration of the synthesis of CILPs. (**B**) TEM image of CILPs. (**C**) DLS curves of LPs, CLPs, ILPs, and CILPs. (**D**) SDS-PAGE determination of the molecular weight of Fc-CV1 (Lane 3) and the relative band brightness of Fc-CV1 in pre-dialyzed CILPs (Lane 1) and dialyzed CILPs (Lane 2) are shown. After being placed in a dialysis bag (300 kDa) and dialyzed against HEPES, only the Fc-CV1, which successfully bound ILPs (~100 nm), could be shown. This step was performed for three rounds in the same way. (**E**) The binding rate of Fc-CV1 inserted on LPs (*n* = 3).

CILPs were successfully prepared by ethanol injection and the ammonium sulfate gradient method. Figure 1A displays the synthesis process of CILPs. A TEM analysis demonstrated that the CILPs were spherical and uniform in shape (Figure 1B). Furthermore, the DLS result revealed that the particle size of the four liposomes was unimodal distribution (Figure 1C). The binding rate (BR) was verified by SDS-PAGE, which is displayed in Figure 1D,E. Figure 1D shows the molecular weight of Fc-CV1, and the relative band brightness of dialyzed and pre-dialyzed CILPs. The BR was calculated to be 67.13 ± 37.10% through Image J’s scanning densitometry of electrophoretic bands (Figure 1E), providing the possibility for CD47-targeting tumor cells.

HPLC was used to detect the encapsulation efficiency (EE) of imiquimod (Supplementary Figure S1). As displayed in Supplementary Figure S1A, the free imiquimod showed a significant single peak, while the CLPs showed no peak under the same test conditions (Supplementary Figure S1B). Therefore, it is feasible to detect the CILPs directly under the test conditions. As expected, the detection values of CILPs before and after dialysis were regarded as the total drug content and the drug content in liposomes, respectively (Supplementary Figure S1C,D). The EE of imiquimod was calculated to be 85.44 ± 4.11%. Compared with the reported passive-loading drug-delivery system [33,34,35], this study achieved higher encapsulation efficiency and a more convenient preparation process.

DLS demonstrated that the diameters of LPs were 111.567 ± 0.655 nm, while CLPs, ILPs, and CILPs showed a slightly larger size of 117.467 ± 0.340 nm, 120.233 ± 0.776 nm, and 130.033 ± 0.974 nm, respectively (Table 1). The changes in the size of these liposomes may be attributed to the modification of Fc-CV1 and imiquimod. The PDI was close to 0.1 (Table 1), indicating that these drug-delivery vehicles were highly homogenous in their distribution. Additionally, the zeta-potential values of LPs, CLPs, ILPs, and CILPs were measured to be −2.231 ± 0.167 mV, −2.492 ± 0.033 mV, −2.383 ± 0.070 mV, and −2.075 ± 0.070 mV, respectively (Table 1), suggesting that the surface-conjugated Fc-CV1 and encapsulated imiquimod had little effect on the charge of liposomes.

### 2.2. In Vitro Cell Uptake Efficiency

**Figure 2:**
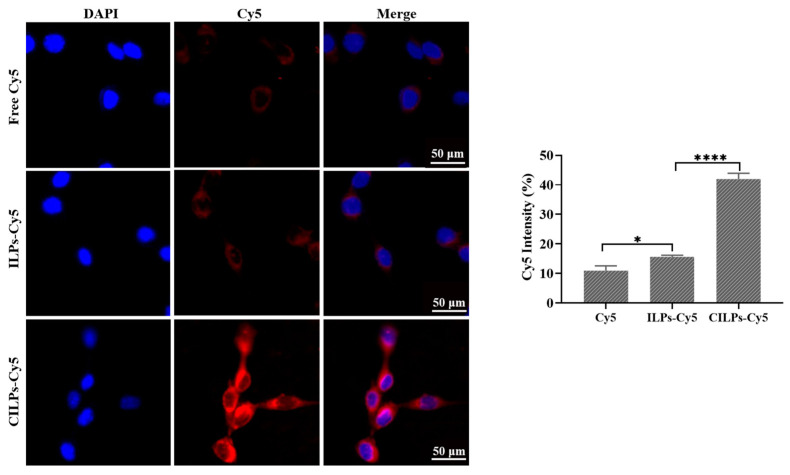
The images of CT26.WT cells after incubation with the free Cy5, ILPs-Cy5, and CILPs-Cy5 (red) for 0.5 h, and staining with DAPI (blue). Scale bar: 50 μm. (*n* = 3). * *p* < 0.05; **** *p* < 0.0001.

The cell uptake efficiency of CILPs was evaluated through the fluorescence intensity of Cy5 taken up by CT26.WT cells using a High-Content Imaging System. As shown in Figure 2, compared with free Cy5 and ILP-Cy5 groups, CILPs-Cy5 groups exhibited a stronger cell uptake capacity after 30 min of incubation. These results inferred that Fc-CV1 enhanced the intracellular delivery ability of imiquimod-loaded liposomes due to the specific affinity to the Fc-CV1 receptor, leading to a more potent anti-tumor effect.

### 2.3. Release Curves of Liposomes

**Figure 3:**
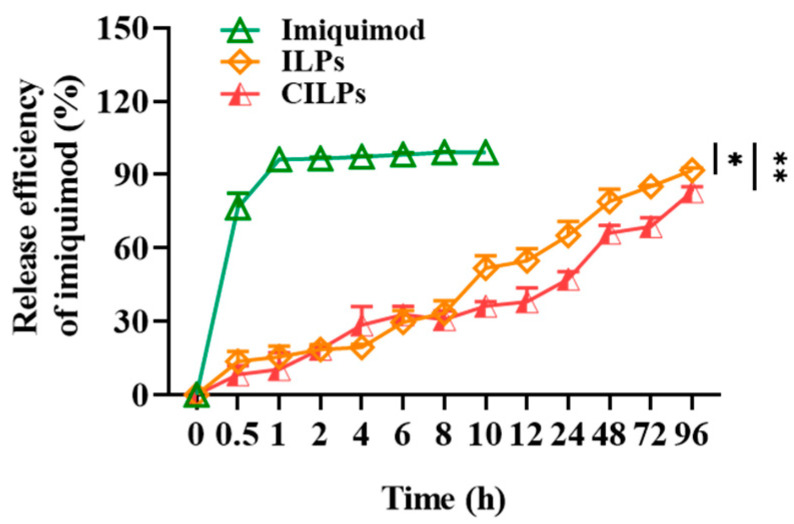
Imiquimod release curves of the free imiquimod, ILPs, and CILPs in PBS at 37 °C. Data are presented as means ± standard deviation (*n* = 3). * *p* < 0.05, ** *p* < 0.01.

The drug release curves of the free imiquimod, ILPs, and CILPs were measured in a simulated in vivo environment. Figure 3 shows that the free imiquimod was released entirely after 1 h, while the release rate (RR) of imiquimod that encapsulated with ILPs and CILPs were only 54.75 ± 4.08% and 39.70 ± 0.05% at 12 h, respectively. Furthermore, the slow release of imiquimod in ILPs and CILPs could be closed with the controlled drug-release property of liposomes. The RR of imiquimod in ILPs and CILPs showed no significant difference at 96 h, indicating that “post insertion” exhibited a negligible impact on the membrane stability of liposomes.

### 2.4. Cytotoxicity Study

**Figure 4:**
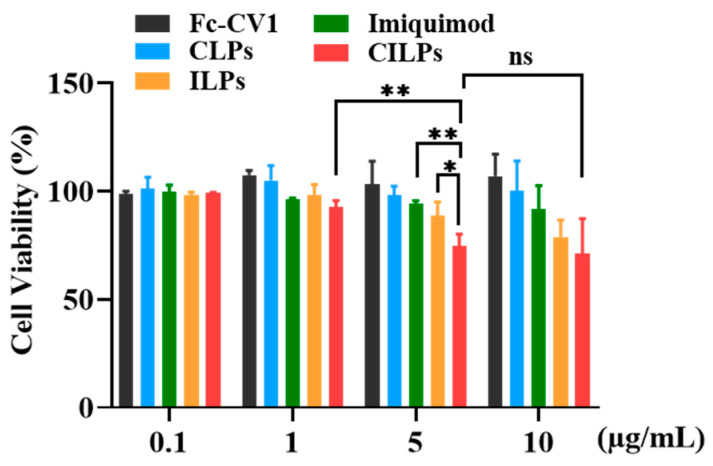
The viability of the cells treated with Fc-CV1, imiquimod, CLPs, ILPs, and CILPs for 24 h. The concentration of the Fc-CV1 and imiquimod were both 0–10 μg/mL. * *p* < 0.05, ** *p* < 0.01, ns = no significant differences.

To study the cytotoxicity of CILPs in vitro, the CCK-8 assay was performed on CT26.WT cells. As shown in Figure 4, CILPs at low concentrations (0.1–1 µg/mL) did not exhibit cytotoxicity, which is consistent with the safety of immunization therapy. Cell viability was slightly decreased after 5 µg/mL imiquimod treatment, probably because of the ROS and endoplasmic reticulum-mediated immunogenic cell death induced by imiquimod [36]. Moreover, CILPs lead to a significant decrease in cell viability compared to control groups, which is attributed to the synergistic effect of imiquimod and Fc-CV1.

### 2.5. Biodistribution Study

**Figure 5:**
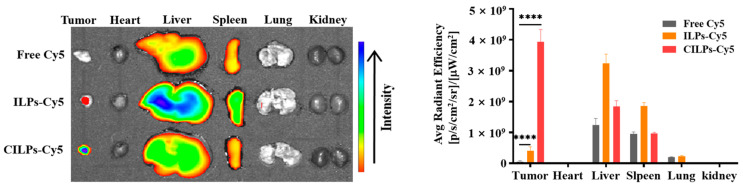
The distribution of Cy5-fluorescence in tumors and major organs at 24 h after intravenous administration. Data are shown as means ± standard deviation (*n* = 3). **** *p* < 0.0001.

The distribution and accumulation of different formulations in tumors and major organs directly affects the therapeutic effect. Following intravenous administration, the fluorescence signals were monitored using a near-infrared vivisection system to assess the distribution and accumulation of different formulations in tumors and major organs after 24 h. As shown in Figure 5 the total fluorescence intensity of ILPs-Cy5 and CILPs-Cy5 was higher than free Cy5, indicating that the liposomes possessed a long-circulating property in vivo. In addition, the fluorescence intensity of the CILPs-Cy5 treatment group was the highest in tumor tissue, which could be ascribed to the tumor-targeting effect of Fc-CV1 ligands, thereby enhancing the anti-tumor effect. Strikingly, the fluorescence intensity of livers and spleens was more potent than other organs, which is probably owing to the phagocytosis of liposomes by the reticuloendothelial system (RES) in the liver and kidney [37].

### 2.6. In Vivo Tumor Inhibition Study

**Figure 6:**
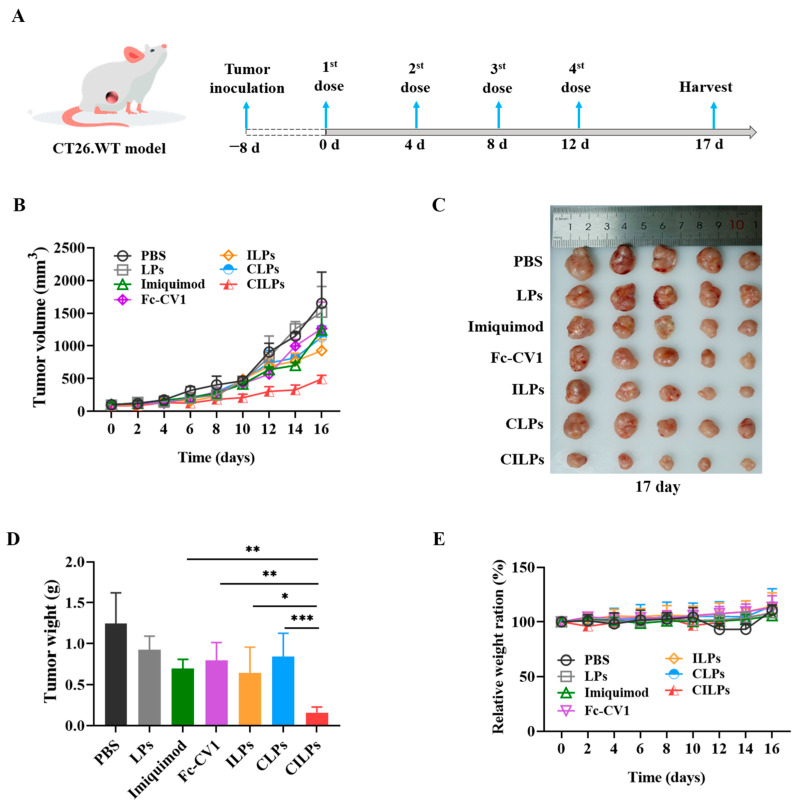
(**A**) Schematic illustration of anti-colon cancer treatment on the CT26.WT tumor model. (**B**) The growth curves of tumor volume during the treatment period. (**C**) Relative tumor photographs and (**D**) weight of the tumor collected from different treatment groups on day 17. (**E**) The relative body weight ratio of the body weight compared with 0 day. Data are presented as mean ± standard deviation (*n* = 5). * *p* < 0.05, ** *p* < 0.01, *** *p* < 0.001.

The therapeutic schedule (Figure 6A) and effects of different preparations are shown in Figure 6. Compared with the PBS and LPs groups, other treatment groups exhibited certain tumor-suppressor effects. Notably, the tumor volume was minimal after treatment with CILPs, suggesting that CILPs possessed an excellent tumor suppressor effect (Figure 6B).

At the end of the experiment, the mice were sacrificed and their tumors were excised, photographed, and weighed to determine the therapeutic efficacy of different preparations. As shown in Figure 6C,D, the treatment group of CILPs had the smallest and lightest tumors compared to other groups, demonstrating that CILPs possessed the greatest tumor-suppressor effects. Table 2 shows that the tumor growth inhibition rate of CILPs was calculated to be 84.35%. In addition, the 84.35% tumor suppression rate was significantly higher than that of the CLPs and ILPs groups, and this means that there is a synergistic effect of the blocking of the “don’t eat me” axis and the activation of the immune response. Compared with the equivalent preparation in vitro (2.5–5 μg/mL), the higher inhibition rate was closely related with that of the formulation activated immune response and blocked the immune escape mechanism in vivo, which was not available in vitro.

### 2.7. CILPs Increased T-Cell Infiltration and Secretion of IFNγ

**Figure 7:**
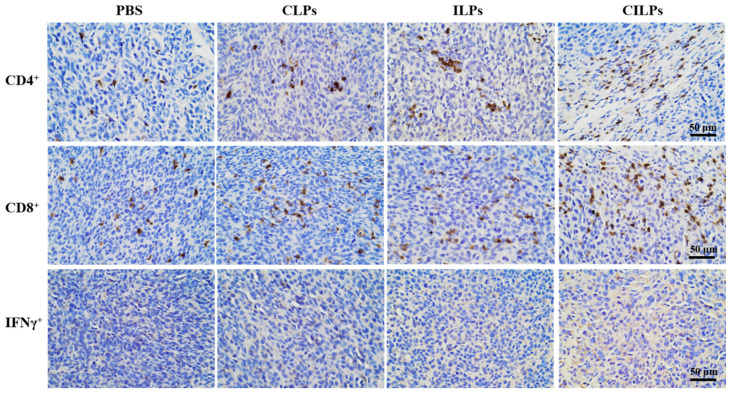
Immunohistochemistry of mouse tumor tissues; positive cells are stained brown. Scale bar, 50 μm (*n* = 5).

To explore the immune cell milieu of the tumors, the immune cell induced by CILPs was analyzed immunohistochemically at the end of the anti-tumor assay. The CD4^+^ and CD8^+^ T cells were increased in the tumor sections of the CILPs groups (Figure 7). The co-delivery of Fc-CV1 and imiquimod therapy resulted in significant CD4^+^ and CD8^+^ T cell infiltration. In addition, there was a significant IFNγ secretion in the tumors undergoing CILPs treatment, which was because that CILPs triggered Toll-like receptor 7 and activated the immune response.

### 2.8. Biosecurity Assessment of CILPs

**Figure 8:**
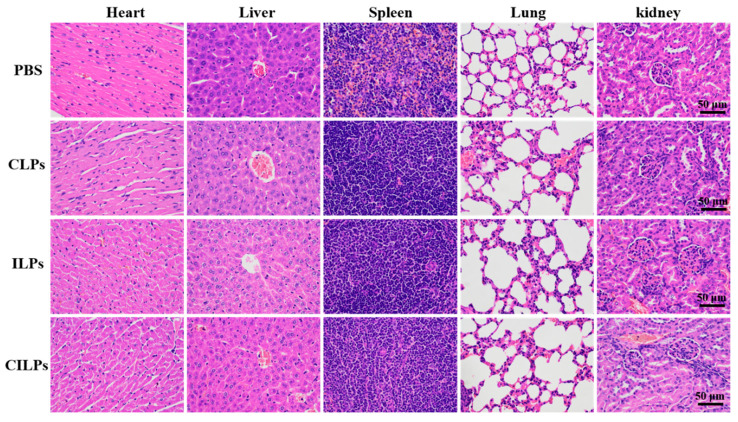
H&E (hematoxylin and eosin) staining of the main organs. Scale bar, 50 μm (*n* = 3).

The biosafety of CILPs-mediated therapy was also an important index in terms of accessing the therapeutic evaluation. During the tumor inhibition study in vivo, CILPs did not induce significant weight loss in the period of treatment (Figure 6D). The biochemical parameters of the liver and kidney were all in the normal range according to the standard reference range of mice (Table 3), and the organ histology showed no significant changes, indicating that the CILPs possessed excellent biosafety (Figure 8). These findings are crucial in assessing the potential of CILPs as a safe and effective immunotherapeutic agent for cancer treatment.

## 3. Materials and Methods

### 3.1. Materials

Imiquimod was purchased from MedChem Express (Shanghai, China). Fc-CV1 was provided by the State Key Laboratory of Antibody Drugs and Targeted Therapy. Cholesterol, hydrogenated lecithin (HSPC), DSPE-PEG_2000_, DSPE-PEG_2000_-NHS, and DSPE-PEG-Cy5 were purchased from the Xi’an Ruixi Biological Technology Co., Ltd. (Xi’an, China). The 4′,6-diamidino-2-phenylindole (DAPI) were obtained from Keygen Biotech (Nanjing, China). Ammonium sulfate was obtained from Sinopharm (Shanghai, China). Methanol (chromatographic grade) and acetonitrile (chromatographic grade) were purchased from the Aladdin Reagent Company (Shanghai, China). The other reagents are all of domestic analytical grade.

Mouse colon cancer cell line CT26. WT was provided by the Cell Bank of the Chinese Academy of Science. The cells were grown in a cell incubator in the RPMI 1640 medium containing 10% FBS (Thermo Fisher Scientific, New York, NY, USA) at 5% CO_2_ and 95% air.

Female BALB/c mice of 4–6 weeks were supplied by the Pengyue Laboratory Animal Breeding Co., Ltd. (Jinan, China).

### 3.2. Preparation of Blank Liposomes

First, 13.455 mg of HSPC, 4.3595 mg of DSPE-PEG_2000_, and 4.7095 mg of cholesterol were precisely weighed and dissolved in 0.1 mL ethanol (HSPC:DSPE-PEG_2000_:cholesterol = 55.5:5.05:39.3 molar ratio). Secondly, the ethanol solution was rapidly injected into a preheated ammonium sulfate solution (250 mM, 1 mL, 65 °C) via a syringe and then incubated for 30 min at 65 °C with magnetic stirring. Subsequently, the prepared solution was successively extruded through the polycarbonate membranes (Avanti, Alabaster, AL, USA); the size was 200 nm, 100 nm, and 50 nm, respectively. Finally, the resulting solution was the blank liposomes (LPs) with a lipid concentration of 22.5 mg/mL.

### 3.3. Preparation of CD47 Targeted Imiquimod-Encapsulated Liposomes

First, DSPE-PEG_2000_-NHS was dissolved in ddH_2_O (60 °C) and then mixed with Fc-CV1 and incubated in a shaking table at room temperature for 5 h (Fc-CV1:DSPE-PEG_2000_-NHS = 12:1 molar ratio). The chemical reaction mechanism of the connection between Fc-CV1 and DSPE-PEG_2000_-NHS involved the NHS ester reacting with the primary amine on Fc-CV1, and formed a table amine bond conjugate. The prepared solution was proportionally mixed with LPs and incubated at 60 °C for 1 h to insert Fc-CV1 into liposomes, which was named “post insertion” [38]. The mixture proportions were that 2.25 mg Fc-CV1 was modified per 22.5 mg liposomes. The CD47 targeted liposomes (CLPs) were then obtained.

Subsequently, CLPs were placed in a dialysis bag (300 kDa) and dialyzed against HEPES (10 mM, pH = 7.4) for 1 h to remove ammonium sulfate and residual Fc-CV1 in the outer aqueous phase. The dialyzed CLPs were incubated with imiquimod solution (5 mg/mL) at a ratio of 1 mg to 7 µmol total phospholipids at room temperature for 24 h and then dialyzed against PBS (300 kDa) three times to eliminate unencapsulated imiquimod, which referred to the ammonium sulfate gradient technique [39]. The CILPs were then obtained.

Additionally, the non-CD47-targeted imiquimod-encapsulated liposomes (ILPs) were prepared. CLPs and ILPs were considered as the control for CILPs.

### 3.4. Characterization

The morphology of these liposomes was photographed with a transmission electron microscope (TEM, Joel, Tokyo, Japan). The size, zeta-potential, and polymer dispersity index (PDI) of these liposomes were determined by dynamic light scattering (DLS) and electrophoretic light scattering (ELS) using the Malvern’s Zeta sizer ZSE (Malvern, UK). The binding rate (BR) of Fc-CV1 inserted to liposomes was measured by 15% sodium lauryl sulfate-polyacrylamide gel electrophoresis (SDS-PAGE) and Image J software (Bethesda, MD, USA). The encapsulation efficiency (EE) of imiquimod was detected by high-performance liquid chromatography (HPLC, Thermo Fisher Scientific, New York, NY, USA).

### 3.5. In Vitro Cell Uptake Study

The in vitro cell uptake of these liposomes was determined by the cumulative fluorescence intensity of Cy5 in the CT26. WT cells. DSPE-PEG-Cy5 was incubated with CILPs and ILPs (DSPE-PEG-Cy5: CILPs or ILPs = 1:5 mass ratio) at 60 °C for 1 h, respectively, and then fluorescent liposomes named as CILPs-Cy5 and ILPs-Cy5 were obtained.

CT26.WT cells were seeded in 96-well plates and randomly divided into three groups (1 × 10^4^ cells/well, *n* = 3). After the cell confluency reached 50–70%, each group was incubated with free Cy5, ILPs-Cy5, and CILPs-Cy5, respectively, for 30 min at 37 °C, in which the final concentrations of Cy5 were 1 µg/mL in 0.1 mL RPMI 1640 medium. Subsequently, the cells were immobilized by 4% paraformaldehyde for 15 min after being washed twice with PBS, and then stained with 10 µL DAPI (1 µg/µL) for 10 min after being washed twice with PBS. Finally, the uptake of each group of cells was recorded by a High-Content Imaging System (Molecular Devices, Sunnyvale, CA, USA) after washing twice with PBS.

### 3.6. The Imiquimod Release Study

The release curves of various drug-loaded liposomes were performed using the dialysis method (300 kDa), and the results were compared with the free imiquimod solution. Briefly, 0.6 mL ILPs, CILPs, and the imiquimod solution were separately packed into the dialysis bags and then placed in 500 mL of PBS solution (pH = 7.4, 37 °C). Equal volumes of samples were extracted from the bags at predetermined time intervals (0, 0.5, 1, 2, 4, 6, 8, 10, 12, 24, 48, 72, and 96 h), and the release rate (RR) of imiquimod was calculated using the following equation:

(1) $$\mathrm{RR}=\left(S_{\mathrm{und}}-S_{d}\right)/S_{\mathrm{und}}\times 100\%$$

where S_und_ and S_d_ represented the HPLC peak area of the imiquimod in pre-dialyzed and dialyzed preparations at various times, respectively (*n* = 3).

### 3.7. The Cytotoxicity of CILPs

CT-26.WT cells were seeded in a 96-well plate (1 × 10^4^/well, *n* = 3) and cultivated in 100 μL of RPMI 1640 medium until the confluency closely reached 70%. In order to determine the cytotoxicity of CILPs, the RPMI 1640 medium was removed from each well and replaced with a complex solution of CILPs and RPMI 1640 medium, with concentrations of Fc-CV1 and imiquimod ranging from 0 to 10 µg/mL. The cells were incubated with a complex solution for 24 h. In addition, the cells which were treated with Fc-CV1, CLPs, imiquimod solution, and ILPs in a similar concentration were considered control groups. The surviving cells were detected with a CCK-8 assay after 24 h, and the viability of the untreated cells was considered 100%.

### 3.8. Biodistribution Study

Nine Female BALB/c mice were injected subcutaneously with CT26. WT cells (5 × 10^5^) in the right flank, and these tumor-bearing mice were randomly divided into three groups (*n* = 3). Tumor volumes were measured using calipers and were calculated by the following formula:

(2) $$\mathrm{Volume}=\mathrm{Length}\times {\mathrm{Width}}^{2}/2$$

When the tumor volume reached 100 mm^3^, free Cy5, ILP-Cy5 and CILPs-Cy5 were injected intravenously at a dose of Cy5 of 0.4 mg/kg. Twenty-four hours after intravenous injection, the tumor, heart, liver, spleen, lung, and kidney were taken out. The fluorescence signals of the tumors and main organs were detected by the IVIS optical imaging system (IVIS Lumina LT III, PerkinElmer, Waltham, MA, USA).

### 3.9. In Vivo Tumor Inhibition Study

Thirty-five female BALB/c mice were randomly divided into seven groups (*n* = 5), and 5 × 10^5^ CT26. WT cells were injected subcutaneously into the right flank of each mouse. When the mouse tumor size was about 100 mm^3^, treatment was initiated. PBS, LPs, imiquimod, Fc-CV1, ILPs, CLPs, and CILPs were intravenously injected on days 0, 4, 8, and 12. The dose of imiquimod was 2.5 mg/kg in the first and second injections, and 5 mg/kg in the third and fourth injections. In all injections, the dose of Fc-CV1 was 5 mg/kg. At this dose, the drug concentration in the body was 2.5–5 μg/mL, which exerted cytotoxicity on cells in an in vitro experiment. Tumor diameters and body weight were measured every day during the treatment. On day 17, the mice’s tumors were taken out and their weights were measured. Tumor growth inhibition (TGI) was calculated according to the following formula:

(3) $$\begin{array}{c} \mathrm{TGI}=\frac{\mathrm{tomor}\;\mathrm{weight}\;\left(\mathrm{experimental}\right)}{\mathrm{tomor}\;\mathrm{weight}\;\left(\mathrm{control}\right)}\times 100\% \end{array}$$

### 3.10. Immunohistochemistry

Immunohistochemistry staining of CD4, CD8, and IFNγ was performed in tumor tissues according to a standard protocol. After deparaffinizing and rehydrating the paraffin section of the sections of tumor tissues, 3% BSA was added to the circle to cover the tissue, and it was then sealed for 30 min at room temperature. The sections were stained with the CD4, CD8, and IFNγ antibodies (Servicebio, Wuhan, China) overnight at 4 °C, and then incubated with a secondary antibody (HRP-labeled, Servicebio, Wuhan, China) at room temperature for 50 min after two washes with PBS. In the end, the sections were visualized under the microscope (Nikon, E100, Tokyo, Japan) after reacting with the DAB chromogenic agent.

### 3.11. Biosecurity Assessment of CILPs

At the end of the tumor-inhibition study, all the tumors were collected, and the weight of these tumors was measured. The biochemical parameters, including alanine transaminase (ALT), aspartate transaminase (AST), creatinine (CREA), and urea (UREA), were detected from the blood of mice. Fresh tumor tissues and major organs were fixed with 4% paraformaldehyde and embedded in paraffin using an embedding machine (Wuhan Junjie Electronics, JB-P5, Wuhan, China). Tissue slices with a thickness of 5 µm were cut through the microtome (Leica Instrument, RM2016, Shanghai, China), and then stained with hematoxylin and eosin (H&E) according to the protocol. Subsequently, the staining slices were observed under a microscope (Nikon, E100, Tokyo, Japan).

### 3.12. Statistical Analysis

All data are expressed as mean ± SD. The significance level between groups was tested by one-way ANOVA using Graphpad Prism 8.0.2. *p* < 0.05 was defined as a significant difference.

### 3.13. Animal Ethics

All procedures and recommendations concerning animals were approved by the Special Committee on Scientific Research Ethics of Liaocheng University following the national guidelines for the care and use of laboratory animals (Approval Code: 2022111010; Approval Date: 1 November 2022).

## 4. Conclusions

In summary, imiquimod-encapsulated CD47-targeted liposomes were successfully developed, and the in vitro results proved that the CILPs possessed a better-sustained release and specific targeting effect. The in vivo results showed that the preparation could be effectively taken up by tumor cells, and that it exhibited excellent tumor treatment efficiency and biosafety due to the dual function of immune response and the innate immune checkpoint blockade. Therefore, imiquimod-encapsulated liposomes coupled to Fc-CV1 seem to be a promising novel nanomedicine to improve CD47 expression tumor treatment. Immunotherapy based on innate immunity may serve as a general strategy against tumor growth for synergistic combinations with ICB in the future.

## Figures and Tables

**Table 1:** The diameters, zeta-potential, PDI, and EE of LPs, CLPs, ILPs, and CILPs, respectively.

Sample	Diameters (nm)	PDI	Zeta-Potential (mV)	EE (%)
LPs	111.567 ± 0.655	0.095 ± 0.005	−2.231 ± 0.167	/
CLPs	117.467 ± 0.340	0.120 ± 0.018	−2.492 ± 0.033	/
ILPs	120.233 ± 0.776	0.125 ± 0.005	−2.383 ± 0.070	81.205 ± 8.102
CILPs	130.033 ± 0.974	0.144 ± 0.011	−2.075 ± 0.070	85.443 ± 4.108

The values are means ± standard deviations (*n* = 3).

**Table 2:** The data of tumor weight and tumor growth inhibition (TGI) of various treatment groups.

Group	Tumor Weight (g)	TGI
PBS	1.246 ± 0.335	\
LPs	0.926 ± 0.150	28.319%
imiquimod	0.697 ± 0.212	44.061%
Fc-CV1	0.799 ± 0.192	35.875%
ILPs	0.643 ± 0.282	48.395%
CLPs	0.846 ± 0.252	32.103%
CILPs	0.195 ± 0.054	84.350%

The values are means ± standard deviations (*n* = 3).

**Table 3:** The biochemical parameters of the liver and kidney.

Groups	ALT (10–96 U/L)	AST (36–235 U/L)	BUN (10–35 mg/dL)	CREA (11–85 umol/L)
PBS	46.75 ± 1.75	262.42 ± 12.42	5.42 ± 0.42	51.34 ± 1.34
ILPs	52.44 ± 2.44	209.58 ± 9.58	7.06 ± 1.06	46.10 ± 1.10
CLPs	78.27 ± 3.27	208.49 ± 8.49	23.12 ± 3.12	39.57 ± 1.57
CILPs	53.36 ± 3.36	232.91 ± 12.91	8.00 ± 1.00	44.87 ± 4.87

Values are the means ± standard deviation (*n* = 3).

## Data Availability

Not applicable.

## Supplementary Materials

The following supporting information can be downloaded at: https://www.mdpi.com/article/10.3390/molecules28073147/s1, Figure S1: HPLC Chromatogram for free imiquimod, CLPs, pre-dialysis CILPs and after-dialysis CILPs.

## Sample Availability

Samples of the Fc-CV1 are available from the authors.
