# Supplementary material for: Liposome-Based Co-Immunotherapy with TLR Agonist and CD47-SIRPα Checkpoint Blockade for Efficient Treatment of Colon Cancer
Source: Molecules. 2023 Mar 31;28(7):3147. doi: 10.3390/molecules28073147 (PMC10095745; doi:10.3390/molecules28073147)
Supplement: Supplementary file 1 [file molecules-28-03147-s001.zip › molecules-2305474-supplementary.pdf]

Supplementary Materials

Binding rate (BR). Half CILPs were dialyzed against PBS (4℃) three times in dialyzed bags to remove the free Fc-CV1, which could be ascribed to the fact that the molecular weight of CILPs was larger than the diameter of dialysis bags (300 kDa) after the insertion of Fc-CV1. After being dialyzed, only the Fc-CV1 that had been successfully linked could be detected by SDS-PAGE. Then, the Fc-CV1, dialyzed, and undialyzed CILPs were subjected to Sodium lauryl sulfate-polyacrylamide gel electrophoresis (SDS-PAGE) with the same mass of Fc-CV1. After that, the undialyzed and dialyzed CILPs were measured based on the densitometry of the SDS-PAGE band by Image J software (Bethesda, MD, USA), and then the BR was obtained by the following formula:

$$BR= (G_{d} / G_{und}) \times 100\%.$$

where G_d and G_{und} represented the grayscale value of dialyzed CILPs of the imiquimod in dialyzed CILPs and undialyzed CILPs, respectively.

Encapsulation efficiency (EE). The CLPs, undialyzed CILPs and dialyzed CILPs were demulsified with chromatographic-grade acetonitrile. Using 100% acetonitrile solution and H2O as mobile phase (acetonitrile: H2O = 1:1, volume/volume) and running at a rate of 1 mL/min, the absorbance of the eluent was determined at 254 nm by Aquasil C18, 150 × 4.6 mm chromatographic column (Thermo Fisher Science, Massachusetts, USA). The EE of imiquimod was calculated by the formula:

$$EE= (S_{d} / S_{und}) \times 100\%$$

where S_d and S_{und} represented the HPLC peak area of the imiquimod in dialyzed CILPs and undialyzed CILPs, respectively.

Characterization of size, zeta-potential, and polymer dispersity index (PDI). For the determination of particle size and PDI, 20 μL of LPs, CLPs ILPs, and CILPs were diluted to 1 mL in PBS solution and determined by the Malvern's Zeta sizer ZSE (Malvern, Worcestershire, UK). The particle size and PDI were measured by the dynamic light scattering method (DLS) because the Brownian motion of the particles suspended in the solution causes the fluctuation of the scattered light intensity. Because the particles move in electrophoresis under the applied electric field, their velocity is directly related to zeta-potential, zeta-potential was measured by the electrophoretic light scattering method (ELS).

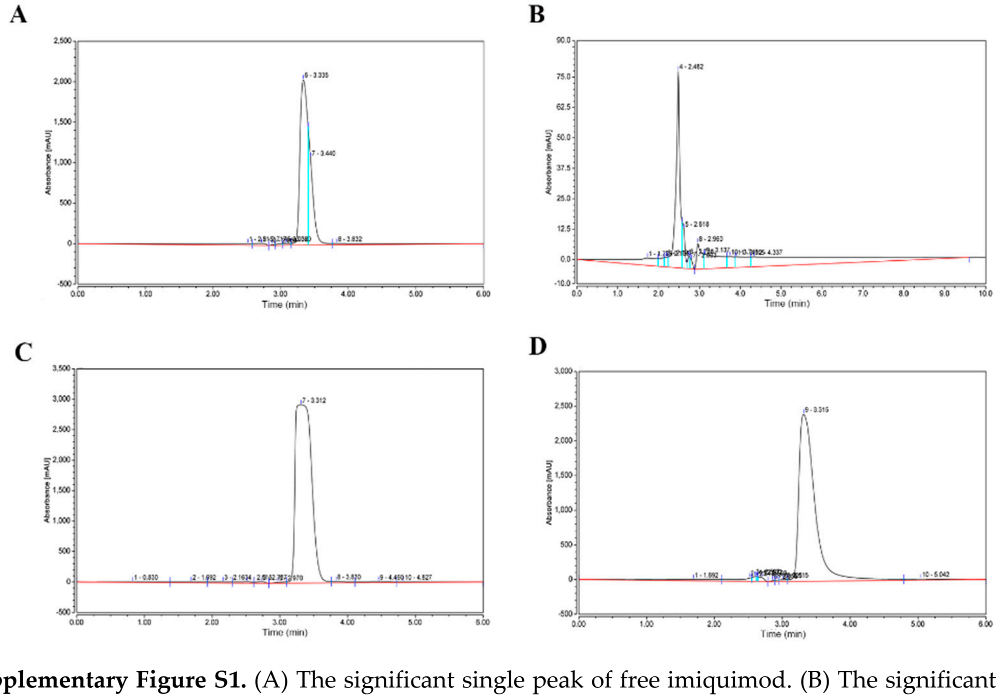

Supplementary Figure S1. (A) The significant single peak of free imiquimod. (B) The significant single peak of CLPs. (C) The significant single peak of CILPs before dialysis. (D) The significant single peak of CILPs after dialysis.
